# If You Don’t Find It Often, You Often Don’t Find It: Why Some Cancers Are Missed in Breast Cancer Screening

**DOI:** 10.1371/journal.pone.0064366

**Published:** 2013-05-30

**Authors:** Karla K. Evans, Robyn L. Birdwell, Jeremy M. Wolfe

**Affiliations:** 1 Visual Attention Lab, Brigham and Women’s Hospital, Harvard Medical School, Cambridge, Massachusetts, United States of America; 2 Radiology, Brigham and Women’s Hospital, Harvard Medical School, Boston, Massachusetts, United States of America; University of Bath, United Kingdom

## Abstract

Mammography is an important tool in the early detection of breast cancer. However, the perceptual task is difficult and a significant proportion of cancers are missed. Visual search experiments show that miss (false negative) errors are elevated when targets are rare (low prevalence) but it is unknown if low prevalence is a significant factor under real world, clinical conditions. Here we show that expert mammographers in a real, low-prevalence, clinical setting, miss a much higher percentage of cancers than are missed when the mammographers search for the same cancers under high prevalence conditions. We inserted 50 positive and 50 negative cases into the normal workflow of the breast cancer screening service of an urban hospital over the course of nine months. This rate was slow enough not to markedly raise disease prevalence in the radiologists’ daily practice. Six radiologists subsequently reviewed all 100 cases in a session where the prevalence of disease was 50%. In the clinical setting, participants missed 30% of the cancers. In the high prevalence setting, participants missed just 12% of the same cancers. Under most circumstances, this low prevalence effect is probably adaptive. It is usually wise to be conservative about reporting events with very low base rates (Was that a flying saucer? Probably not.). However, while this response to low prevalence appears to be strongly engrained in human visual search mechanisms, it may not be as adaptive in socially important, low prevalence tasks like medical screening. While the results of any one study must be interpreted cautiously, these data are consistent with the conclusion that this behavioral response to low prevalence could be a substantial contributor to miss errors in breast cancer screening.

## Introduction

Mammographic screening is an important tool in the early detection of breast cancer [1] but it is a difficult perceptual task and error-prone [2] with reported false negative rates of 20–30% [3], [4]. The signs of breast cancer are often ambiguous and/or hard to see, with some proportion of errors attributable to the perceptual difficulty of the task. However, a significant proportion of miss errors cannot be attributed to a lack of a clear signal. In many cases, if disease is detected in the current exam, it can also be seen in retrospect on the previous exam. These “retrospectively visible” or “actionable” cancers could have been found but were missed on that previous exam [5]–[7]. They are either errors in perception (failures of search) [8], or alternatively errors in interpretation. Here we consider one contributor to those failures, namely the low prevalence of disease in screening mammograms.

Breast cancer screening by mammography is a difficult visual search task, characterized by a low prevalence of positive findings. Experiments with non-experts in a laboratory setting show that more targets are missed during vigilance tasks when observers monitor displays for targets that appear infrequently [9], [10]. Attention fluctuates and targets can come and go without being noticed. More recently, it has been shown that these prevalence effects occur in visual search tasks even though observers can view displays for as long as they want. Even when observers must actively reject a display before it will be removed, more targets are missed at low prevalence than at higher prevalence [11].

The opposite type of error, false positives, tend to decline at low prevalence [12] because the primary effect of prevalence is a criterion shift, with observers in low prevalence situations less likely to call an ambiguous stimulus a target and more likely to terminate search [13]. In clinical settings, neither false positives nor false negative errors are desirable but it seems reasonable to assert that false negative errors are less desirable. Thus, if low prevalence produces more false negative errors in a clinical setting, even if the false positive errors decline, that would be important information.

It is important to note that prevalence effect could have two different types of effect on performance in mammography [14]–[16]. Gur and his colleagues have shown that, in a laboratory setting, prevalence did not change the area under the receiver operating characteristic curve (AUC) [14]. However, even if AUC is unchanged, it would be of interest to find the change in the pattern of errors that would follow a change in criterion, the bias to call a case actionable or non-actionable. In reanalyzing the 2003 data, Gur et al. (2008) reported a change in confidence ratings with prevalence that would be consistent with a criterion shift and, as noted above, criterion shifts have been a hallmark of prevalence effects outside radiology [15]. Our particular interest was in looking for evidence for a prevalence effect in the clinic with professionals carrying out a critical task in their area of expertise.

There is evidence that prevalence is a factor for experts in cervical cancer screening [17], a situation that, like mammography, is characterized by a low prevalence of disease in the tested population (estimated to be about 0.3%) [18]–[20]. The present study was designed to compare error rates in mammography under low prevalence, clinical conditions with rates under high prevalence, laboratory conditions. Doing a study of this sort under realistic clinical conditions is conceptually easy but difficult to implement because of the need to minimize interference with the normal clinical workload and the need to insert test cases undetectably without violating patient rights. In order to measure performance under true clinical conditions, 100 cases were selected by study radiologist (RB): 50 with biopsy-confirmed cancers and 50 determined to be negative based on two to three years of stable negative findings. We introduced these cases into the offline, regular screening workflow over a 9-month time period (i.e. radiologists were reading these screening mammograms in a batch a few hours or few days after their acquisition). We used this very slow trickle of cases so as not to seriously alter the overall prevalence of disease in this screening population. In the second arm of the study, all of the same 100 cases were interpreted in a single sitting by members of the same group of participating radiologists.

Ideally, we would have tested high and low prevalence under conditions that differed only in prevalence. Obviously, that is not the case in this study where low prevalence cases were unobtrusively slipped into the normal workflow while high prevalence cases were read under laboratory conditions. Unfortunately, the ideal version of this experiment is impossible. If the high prevalence arm of the study was generated by adding cases into clinical practice in order to boost prevalence to 50% or even some much more modest level (e.g. 10%), clinicians would know immediately that this was not normal clinical practice in a world where cancer prevalence is normally about 0.3%. Nor can the low prevalence arm be run under laboratory conditions. First, that would lose the realism of testing in the clinic. Moreover, it would be prohibitive in the lab to read the thousands of normal cases that would be needed in order to have prevalence near 1%. The present design is a compromise between the ideal and the possible. It maintains the basic goal of reading the same 100 cases under high and low prevalence conditions. We will return to these concerns in discussing the results.

Within the constraints imposed by the real world, we show that false negative errors are higher in the low prevalence clinical setting than in the high prevalence, lab setting, suggesting that a substantial portion of missed cancers may be missed because of the properties of the human ‘search engine’.

## Results

We measured false negative and false positive rates in both the high and low prevalence settings. Because we could not control which radiologist saw which case in the low prevalence arm, data from the low prevalence arm was treated as if the entire 14-radiologist practice constituted one experimental observer. No radiologist in the low prevalence arm reported recognizing that an inserted case was not a part of the normal workflow. Inclusion of inserted cases in the low prevalence arm of the study raised disease prevalence from ∼0.3% to ∼1% during the study period, a change unlikely to influence prevalence effects^5^. These low prevalence data were compared to the average performance of 6 observers that also participated in the high prevalence condition. These 6 radiologists contributed 41% of the low prevalence interpretations (see below).

**Figure 1:**
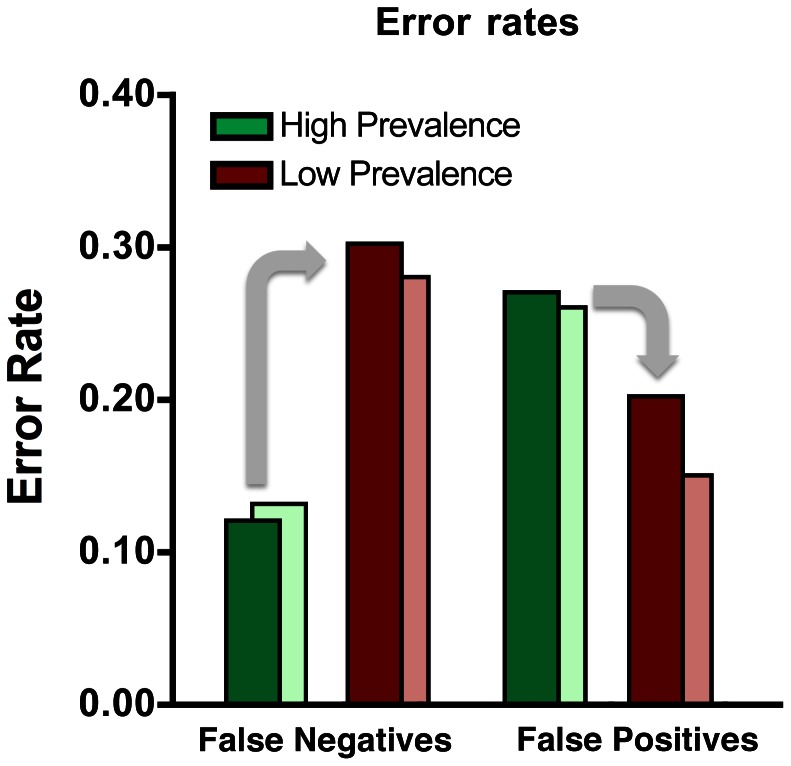
Error rates for rare targets (red bars, ∼1% prevalence) and common targets (green bars, 50% prevalence) for two types of errors, false negatives and false positives. The dark colored bars represent data average over all 14 observers. The light red bars represent low prevalence average errors (false negatives and false positives) for the six observers who participated in both arms of the study (low and high prevalence). The light green bars represent high prevalence average errors (false negatives and false positives) restricted to the cases that the six high prevalence observers did not also see during the low prevalence arm of the study. Regardless of these filtering of the data, low prevalence, false negative errors are markedly higher than high prevalence false negative errors.

We compared performance of the radiologists in the low and high prevalence reading settings only after removing from high prevalence analysis any case that a radiologist saw in both arms of the study (though, in fact, this does not change the pattern of results). As shown in Figure 1, the false negative rate was 12% at high prevalence, rising significantly to 30% at low prevalence for the same set of the 50 positive cases (χ^2^_(1)_ = 11.77, p<0.005). False positives were lower at low prevalence, though the difference is not significant (Low prevalence: 20%; High prevalence: 27%; χ^2^_(1)_ = 3.04, p>0.05).

**Figure 2:**
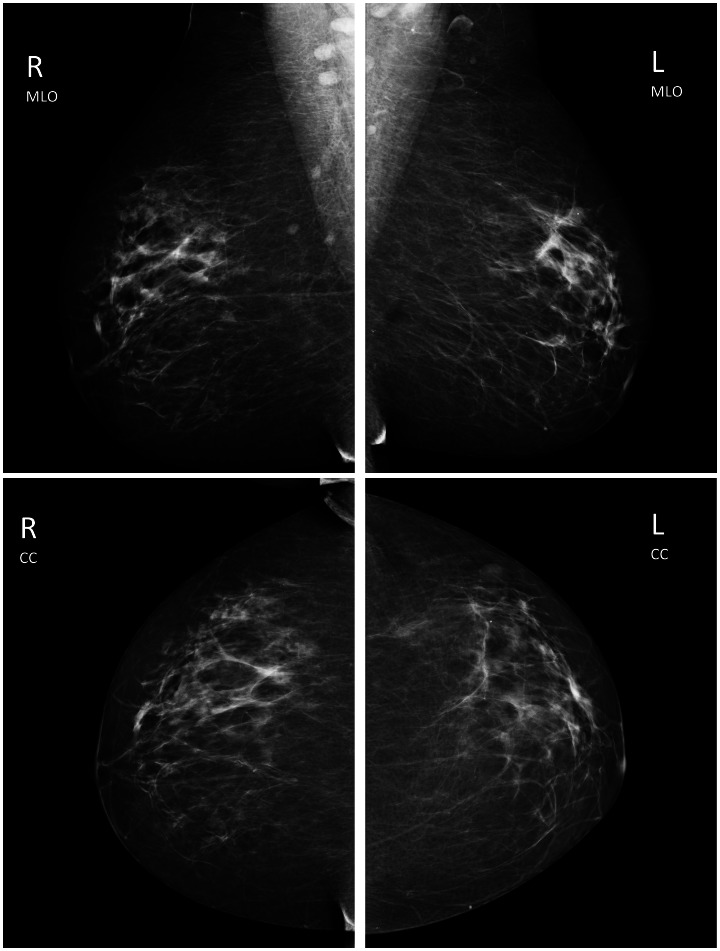
One of the seven cases not seen at low prevalence but seen by all 6 readers at high prevalence. This is a case of a 56-year-old woman whose test screening mammogram was presented with a prior mammogram taken two years earlier. The case was rated level 5 of difficulty, the cancer was detected on the original screening and the lesion type is calcifications measuring 10 mm in size with the pathology of DCIS with microinvasion. The parenchymal density is less dense. a) MLO (top 2 images) and CC (bottom two images). b) Lesion in right upper quadrant. Magnification view shows pleomorphic calcifications in a segmental distribution.

Of 15 cancers missed in low prevalence, seven (47%) were found by all 6 observers that also participated in the high prevalence arm of the study (Figure 2). Eight of the remaining cancers missed at low prevalence were detected by at least one radiologist at high prevalence. The pattern of significantly higher false negatives and lower false positives during low target prevalence compared to high target prevalence does not change if analysis of low prevalence data is restricted to the 41% of cases interpreted by the six radiologists who participated in both arms of the study, though statistical power declines (shown as pale bars of Figure 1).

## Discussion

In mammography, cancers are missed for many reasons. Based on the present results, we propose that the low prevalence of disease is, itself, a source of misses. The same cancers that are found when cancers are present on 50% of cases, are missed when cancers are present on 1% of cases. Given the other differences between the high and low prevalence arms of this study, it is possible to argue that some factor, other than prevalence produced the difference in the pattern of errors. That factor would need to be a factor that provoked a higher miss rate when the clinician/observers believed that they were doing their real job and a lower miss rate when they were doing an experiment. It is unlikely that motivation accounts for the difference between low and high prevalence performance. If anything, we would expect motivation to have been higher when apparently real patients were being evaluated in the low prevalence arm of the study compared to the obviously unreal, laboratory setting of high prevalence. Moreover, the pattern of results closely mirrors other prevalence effects in visual search. The miss (false negative) error rate goes up. The false positive rate declines (albeit by a non-significant amount in this study).

Under most natural circumstances, the effects of low prevalence effect are probably adaptive. It is usually wise to be conservative about reporting events with very low base rates. However, while this response to low prevalence appears to be strongly engrained in human visual search mechanisms, it may not be adaptive in socially important, low prevalence tasks like medical screening.

Of course, prevalence is not the only source of false negative errors. Other causes include failures of perception and/or the analysis of perceived findings [21]. Clearly, technical problems such as poor compression and positioning may lead to inability to see and characterize lesions. Other errors can be attributed to specific parenchymal patterns and lesion location: dense tissue with lesion obscuration, location of lesion near edge of tissue or edge of image, large breasts, and breasts with multiple findings; as well as to challenges more specific to lesion type: subtle malignant features, slow lesion growth, and small lesion size [4].

In this study, we add low prevalence to the list of causes. Most of the cancers that were missed at low prevalence, were found at high prevalence by all or at least one of the study radiologists. The radiologists did not report remembering the cases and there are data that suggest that it is unlikely that the cases would be remembered when reread by the same radiologists [22]–[25]. Moreover, as noted above, the results are essentially the same if we remove from analysis any data from a high prevalence case if the observer had seen that case at low prevalence. This experiment strongly suggests that a substantial prevalence effect can be found outside the lab, when experts are performing important visual search tasks under their normal working conditions. We included a wide range of lesion types and sizes but did not find evidence that the prevalence effect was limited to specific types of lesion or size. Small numbers limit any significant analysis, however positive cases missed at low prevalence but found by all (7 cancers) versus cases missed by at least one reader (8 cases) at high prevalence were rated more often at difficulty levels of 4 and 5 (86% vs. 38%) and had less instances of dense breast parenchyma (43% vs. 63%) (see Tables S1, S2 and S3 for more details).

As noted earlier, there are inevitable compromises in an experiment that wants to examine the effects of low prevalence in a clinical setting. Because, workflow demands meant that we could not guarantee that a specific radiologist would see a specific inserted case, we treated the entire practice as a single observer in the low prevalence arm of the study. Ideally, the same observers would have seen the same number of cases in both arms of the study. This could not be done without disrupting ongoing clinical work. Nevertheless, to the best of our knowledge, this is the only study of prevalence where cases with ground truth were evaluated under normal working conditions at low prevalence. Another limitation of the design is that the comparison is done between a clinical setting and a laboratory setting in which finding a cancer would not alter management. However, as mentioned earlier, it is impossible to test at high prevalence in a clinical screening setting. In a future study, it might be valuable to compare performance on cases inserted into screening to performance on cases inserted at secondary evaluation stage where the real and perceived prevalence of disease would be much higher, though that would pose issues of its own.

Our method for inserting known cases into the regular workflow has potential to be a part of a quality assessment audit system. For the present study, this was a challenging ad hoc process with fictitious names and numbers generated, linked to images, with appropriate history and records and then blocked from any official interpretation. However, if made into a routine practice, this method could provide a relatively unobtrusive mode for individual and/or group assessment. Such a method, using cases where ‘gold standard’ truth is known, could address some of the variability in estimates of rates of missed cancers. For example, in the retrospective analysis, there are differences among cancers that are deemed “missed” that depend on whether or not the reviewer is blinded to the later positive case at the time they assess the earlier “negative” case with miss rates ranging from 10 to as high as 35% [6], [7].

In summary the results illustrate how unanticipated consequences can arise when civilization designs an artificial but important visual search task like breast cancer screening or, for that matter, airport baggage screening, bridge fatigue examinations, etc. The present results indicate that the normal response of the human mind to low probability events could be a substantial contributor to false negative errors in breast cancer screening. What can be done? General methods suggested to improve performance include training, experience, continuing education, prospective double reading, retrospective evaluation of missed cases, or computer-aided detection, and at least in some populations, the use of digital rather than film-screen imaging [19], [26]. In the specific case of prevalence effects, in the laboratory, it is possible to manipulate these effects, for example, by presenting an observer with a burst of high prevalence images prior to low prevalence search [12]. Given the present results, we should now determine if there are practical changes in clinical settings that can reduce errors due to target prevalence.

## Materials and Methods

### Study Participants

Fourteen board certified radiologists with expertise in breast imaging (5 to 30 years of experience) in a large academic hospital, actively engaged in the daily practice of breast-image screening agreed to participate in the study. This prospective study was reviewed and approved by Brigham and Women’s Hospital Institutional Review Board and was HIPPA compliant. All participants gave written informed consent to participate in a prospective study and a subgroup of participants agreed to participate in a later retrospective laboratory study. We have complied according to the principles expressed in the Declaration of Helsinki in the treatment of our participants.

### Study Material

The 100 mammograms (50 positive, 50 negative) used in both phases of the study were acquired with GE digital mammography equipment and presented on the GE Seno Advantage 2.1 workstation in DICOM format. All the cases included at least 4 images (left and right breast mediolateral oblique (MLO) views and craniocaudal (CC) views) and 46% of them included historical prior mammograms for comparison. When selecting cases the study radiologist made an effort to include different tumors types (e.g. masses, calcifications, etc.) and different levels of subjectively assessed difficulty. CAD overlays were used. Header files of the images were populated with fictitious names, dates of birth, requisition number and any other information that might point to the real identity of the patient. True year of birth was preserved. Fictitious unique identifier numbers were created for all cases. Only if participating radiologists attempted to enter a final abnormal report, were they informed that this was an inserted case.

The 50 positive mammograms were either screen-detected cancers, confirmed with later biopsy, or mammograms done 1 to 2 years prior to a screen-detected cancer that had been interpreted as negative. In these latter cases, the lesion was determined to be retrospectively visible by the study radiologist (see Tables S1, S2 and S3 for details about these cases). The other fifty cases of the 100 study cases used had no evidence of cancer for at least 2 years beyond the mammograms that were chosen for the study and were thus characterized as normal. For present purposes, the most important point is that the same 100 cases were seen in low and high prevalence arms of the study.

### Procedure

During both the low and high prevalence study phases, radiologists evaluated one case at a time. They decided whether to recommend further evaluation or not using the standard methods of reporting, and indicated the location of lesions, if present, on the image. In the low prevalence setting (∼1%), each of the 100 study cases was added surreptitiously to the normal off-line screening workflow. Each case was added only once to the normal flow and, therefore, interpreted by only one of the 14 participants. Each of the 14 radiologists saw on average eight of the test cases. In the high prevalence arm of the study that took place nine months after the end of the low prevalence arm, six of the fourteen radiologists each interpreted all 100 test cases in a laboratory setting over approximately three hours. In this arm, prevalence was 50% and observers necessarily knew that they were in a study and were not carrying out their clinical duties. The selection of six radiologists for this arm of the study was random and based on their availability without prior knowledge of their performance in the low prevalence arm of the study.

### Data Analysis

The main outcome measures were false negative and false positive rates as a function of target prevalence. False negative cases were considered those with visible cancers, reported as negative. The false positives were those known-negative mammograms reported as abnormal. The data were analyzed for statistical significance using the chi-squared test.

## Supporting Information

Table S1 **Characteristics of all 50 positive cases used in both arms of the study.**
(DOCX)

Table S2 **Characteristics of the 7 positive cases that were missed in low prevalence arm of the study and fond by all observers in the high prevalence arm of the study.**
(DOCX)

Table S3 **Characteristics of 8 positive cases that were missed in the low prevalence arm of the study and found by at least one observer in the high prevalence arm of the study.**
(DOCX)
