# Supplementary material for: If You Don’t Find It Often, You Often Don’t Find It: Why Some Cancers Are Missed in Breast Cancer Screening
Source: PLoS One. 2013 May 30;8(5):e64366. doi: 10.1371/journal.pone.0064366 (PMC3667799; doi:10.1371/journal.pone.0064366)
Supplement: Table S1 — Characteristics of all 50 positive cases used in both arms of the study. (DOCX) [file pone.0064366.s001.docx]

Table 1. Characteristics of all 50 positive cases used in both arms of the study.

| **Age at Screening Mammogram** | **Study reader had examinations for comparison** | **Subjective Difficulty Rating** | **Lesion Type** | **Lesion Size** | **Cancer Originally Detected**  **(YES, NO)** | **Pathology** | **Parenchymal Density** |
| --- | --- | --- | --- | --- | --- | --- | --- |
| 71 | NO | 3 | CALCIFICATION | 35 mm | YES | IDC, DCIS | MORE DENSE |
| 64 | NO | 2 | MASS, LOBULAR & SMOOTH | 20 x 15 mm | NO | IDC, DCIS | LESS DENSE |
| 64 | NO | 4 | CALCIFICATION | 6 mm | YES | DCIS | LESS DENSE |
| 63 | ONE YEAR EARLIER | 1 | MASS ILL DEFINED | 35 mm | NO | ILC | MORE DENSE |
| 70 | THREE YEARS EARLIER | 2 | CALCIFICATION | 14.5 mm | YES | DCIS | LESS DENSE |
| 64 | ONE YEAR EARLIER | 3 | MASS OVAL & SMOOTH | 8 mm | NO | IDC, DCIS | MORE DENSE |
| 61 | NO | 3 | MASS, IRREGULAR | 11 mm | YES | IDC, DCIS | LESS DENSE |
| 62 | NO | 4 | ASYMMETRY | 9 mm | NO | IDC, | MORE DENSE |
| 70 | NO | 3 | MASS LOBULAR & IRREGULAR | 9 mm | YES | IDC, DCIS | MORE DENSE |
| 62 | NO | 1 | ASYMMETRY | 10 mm | YES | IDC | LESS DENSE |
| 66 | NO | 4 | CALCIFICATION; MASS IRREGULAR & DISTINCT | 8.7 mm | YES | DCIS WITH MICROINVA-SION | LESS DENSE |
| 63 | NO | 3 | CALCIFICATION | 7 mm | YES | IDC | MORE DENSE |
| 62 | NO | 4 | CALCIFICATION | 4 mm | NO | IDC, DCIS | MORE DENSE |
| 38 | NO | 5 | FOCAL ASYMMETRY | 12 mm | YES | IDC, DCIS | MORE DENSE |
| 60 | NO | 2 | MASS IRREGULAR; CALCIFICATION | 17 mm | YES | IDC, DCIS | LESS DENSE |
| 46 | NO | 3 | CALCIFICATION | 5 mm | YES | DCIS | MORE DENSE |
| 41 | NO | 1 | CALCIFICATION | 17 mm | YES | IDC, DCIS | MORE DENSE |
| 57 | NO | 1 | MASS OVAL & SPECULATED; CALCIFICATIONS | 16 mm | YES | IDC, DCIS | MORE DENSE |
| 57 | NO | 3 | MASS OVAL & INDISTINCT | 6 mm | YES | IDC, DCIS | LESS DENSE |
| 65 | NO | 3 | MASS IRREGULAR | 20 mm | YES | IDC, DCIS | LESS DENSE |
| 57 | NO | 4 | MASS ROUND & IRREGULAR | 7 mm | YES | IDC, DCIS | MORE DENSE |
| 47 | NO | 5 | ASYMMETRY | 4 mm | NO | IDC, DCIS | MORE DENSE |
| 74 | NO | 5 | FOCAL ASYMMETRY | 13 mm | NO | INVASIVE WITH MIXED FEATURES | MORE DENSE |
| 75 | THREE YEARS EARLIER | 3 | MASS IRREGULAR | 17 mm | YES | INVASIVE WITH MIXED FEATURES | MORE DENSE |
| 70 | TWO YEARS EARLIER | 5 | MASS ROUND & INDISTINCT | 6 mm | YES | IDC, DCIS | LESS DENSE |
| 45 | ONE YEAR EARLIER | 1 | CALCIFICATION | 30 mm | YES | IDC, DCIS | MORE DENSE |
| 68 | ONE YEAR EARLIER | 4 | ARCHITECTUAL DISTORTION | 15 mm | YES | IDC, DCIS | LESS DENSE |
| 56 | TWO YEARS EARLIER | 5 | CALCIFICATION | 10 mm | YES | DCIS WITH MICOR-INVASION | LESS DENSE |
| 77 | TWO YEARS EARLIER | 5 | CALCIFICATION | 11 mm | NO | DCIS | LESS DENSE |
| 76 | NO | 1 | MASS | 17 mm | YES | IDC, DCIS | LESS DENSE |
| 64 | ONE YEAR EARLIER | 3 | MASS IRREGULAR | 15 mm | NO | INVASIVE WITH MIXED FEATURES, DCIS | MORE DENSE |
| 45 | NO | 1 | CALCIFICATION | 15 mm | YES | DCIS WITH MICRO-INVASION | MORE DENSE |
| 66 | ONE YEAR EARLIER | 4 | MASS OVAL & IRREGULAR | 27 mm | YES | IDC, DCIS | MORE DENSE |
| 63 | TWO YEARS EARLIER | 5 | ASYMMETRY | 16 mm | NO | INVASIVE MIXED FEATURES, DCIS | LESS DENSE |
| 58 | ONE YEAR EARLIER | 3 | ASYMMETRY | 13 mm | NO | ILC | MORE DENSE |
| 69 | NO | 4 | FOCAL ASYMMETRY | 6 mm | YES | IDC | LESS DENSE |
| 55 | TWO YEARS EARLIER | 4 | CALCIFICATION | 9 mm | YES | DCIS | MORE DENSE |
| 52 | ONE & TWO YEARS EARLIER | 2 | ARCHITECTUAL DISTORTION | 20 mm | YES | IDC, DCIS | LESS DENSE |
| 67 | NO | 1 | CALCIFICATION | 13 mm | YES | DCIS | LESS DENSE |
| 39 | ONE YEAR EARLIER | 5 | ASYMMETRY | 10 mm | NO | INVASIVE MIXED FEATURES, DCIS | MORE DENSE |
| 67 | ONE & TWO YEARS EARLIER | 1 | CALCIFICATION | 15 mm | YES | IDC, DCIS | MORE DENSE |
| 48 | FIVE YEARS EARLIER | 2 | CALCIFICATION | 12 mm | YES | DCIS | LESS DENSE |
| 77 | NO | 3 | 2 MASSES OVAL & IRREGULAR | 23 mm | NO | IDC, DCIS | LESS DENSE |
| 77 | ONE & TWO YEARS EARLIER | 2 | MASS OVAL & IRREGULAR | 12 mm | NO | IDC, DCIS | LESS DENSE |
| 43 | TWO YEARS EARLIER | 1 | CALCIFICATION | 48 mm | YES | IDC, DCIS | MORE DENSE |
| 68 | TWO YEARS EARLIER | 3 | CALCIFICATION | 4 mm | YES | DCIS | LESS DENSE |
| 57 | ONE YEAR EARLIER | 4 | CALCIFICATION | 3 mm | NO | DCIS | LESS DENSE |
| 68 | ONE YEAR EARLIER | 3 | FOCAL ASYMMETRY | 6 mm | NO | IDC, DCIS | MORE DENSE |
| 56 | ONE & TWO YEARS EARLIER | 4 | ASYMMETRY | 6 mm | YES | IDC | MORE DENSE |
| 64 | ONE YEAR EARLIER | 2 | ASYMMETRY | 15 mm | NO | ILC AT TWO SIDES | MORE DENSE |
