# Supplementary material for: If You Don’t Find It Often, You Often Don’t Find It: Why Some Cancers Are Missed in Breast Cancer Screening
Source: PLoS One. 2013 May 30;8(5):e64366. doi: 10.1371/journal.pone.0064366 (PMC3667799; doi:10.1371/journal.pone.0064366)
Supplement: Table S2 — Characteristics of the 7 positive cases that were missed in low prevalence arm of the study and fond by all observers in the high prevalence arm of the study. (DOCX) [file pone.0064366.s002.docx]

Table 2. Characteristics of the 7 positive cases that were missed in low prevalence arm of the study and fond by all observers in the high prevalence arm of the study.

| **Age at Screening Mammogram** | **Study reader had examinations for comparison** | **Subjective Difficulty Rating** | **Lesion Type** | **Lesion Size** | **Cancer Originally Detected**  **(YES, NO)** | **Pathology** | **Parenchymal Density** |
| --- | --- | --- | --- | --- | --- | --- | --- |
| 64 | NO | 4 | CALCIFICATION | 6 mm | YES | DCIS | LESS DENSE |
| 62 | NO | 4 | CALCIFICATION | 4 mm | NO | IDC, DCIS | MORE DENSE |
| 75 | THREE YEARS EARLIER | 3 | MASS IRREGULAR | 17 mm | YES | INVASIVE WITH MIXED FEATURES | MORE DENSE |
| 56 | TWO YEARS EARLIER | 5 | CALSIFICATION | 10 mm | YES | DCIS WITH MICOR-INVASION | LESS DENSE |
| 77 | TWO YEARS EARLIER | 5 | CALSIFICATION | 11 mm | NO | DCIS | LESS DENSE |
| 57 | ONE YEAR EARLIER | 4 | CALSIFICATION | 3 mm | NO | DCIS | LESS DENSE |
| 56 | ONE & TWO YEARS EARLIER | 4 | FOCAL ASYMMETRY | 6 mm | YES | IDC | MORE DENSE |
