# Supplementary material for: If You Don’t Find It Often, You Often Don’t Find It: Why Some Cancers Are Missed in Breast Cancer Screening
Source: PLoS One. 2013 May 30;8(5):e64366. doi: 10.1371/journal.pone.0064366 (PMC3667799; doi:10.1371/journal.pone.0064366)
Supplement: Table S3 — Characteristics of 8 positive cases that were missed in the low prevalence arm of the study and found by at least one observer in the high prevalence arm of the study. (DOCX) [file pone.0064366.s003.docx]

Table 3. Characteristics of 8 positive cases that were missed in the low prevalence arm of the study and found by at least one observer in the high prevalence arm of the study.

| **Age at Screening Mammogram** | **Study reader had examinations for comparison** | **Subjective Difficulty Rating** | **Lesion Type** | **Lesion Size** | **Cancer Originally Detected**  **(YES, NO)** | **Pathology** | **Parenchymal Density** |
| --- | --- | --- | --- | --- | --- | --- | --- |
| 63 | NO | 3 | CALCIFICATION | 7 mm | YES | IDC | MORE DENSE |
| 57 | NO | 4 | MASS ROUND AND IRREGULAR | 7 mm | YES | IDC, DCIS | MORE DENSE |
| 63 | TWO YEARS EARLIER | 5 | ASYMMETRY | 16 mm | NO | INVASIVE MIXED FEATURES, DCIS | LESS DENSE |
| 58 | ONE YEAR EARLIER | 3 | ASYMMETRY | 13 mm | NO | ILC | MORE DENSE |
| 39 | ONE YEAR EARLIER | 5 | ASYMMETRY | 10 mm | NO | INVASIVE MIXED FEATURES, DCIS | MORE DENSE |
| 77 | ONE & TWO YEARS EARLIER | 2 | MASS OVAL IRREGULAR | 12 mm | NO | IDC, DCIS | LESS DENSE |
| 68 | TWO YEARS EARLIER | 3 | CALCIFICATION | 4 mm | YES | DCIS | LESS DENSE |
| 68 | ONE YEAR EARLIER | 3 | FOCAL ASYMMETRY | 6 mm | NO | IDC, DCIS | MORE DENSE |
